# Dental amalgam and urinary mercury concentrations: a descriptive study

**DOI:** 10.1186/1472-6831-13-44

**Published:** 2013-09-09

**Authors:** Alexandra Nicolae, Harry Ames, Carlos Quiñonez

**Affiliations:** 1 Community Dental Health Services Research Unit, University of Toronto, 124 Edward Street, M5G 1G6, Toronto, ON, Canada; 2 Alberta Dental Association and College, Suite 101, 8230-105 Street NW, T6E 5H9, Edmonton, AB, Canada

**Keywords:** Dental amalgam, Mercury, Urinary levels

## Abstract

**Background:**

Dental amalgam is a source of elemental and inorganic mercury. The safety of dental amalgam in individuals remains a controversial issue. Urinary mercury concentrations are used to assess chronic exposure to elemental mercury. At present, there are no indications of mercury-associated adverse effects at levels below 5 μg Hg/g creatinine (Cr) or 7 μg Hg/L (urine). The purpose of the present study is to determine the overall urinary mercury level in the Canadian general population in relation to the number of dental amalgam surfaces.

**Methods:**

Data come from the 2007/09 Canadian Health Measures Survey, which measured urinary mercury concentrations in a nationally representative sample of 5,418 Canadians aged 6–79 years. Urinary mercury concentrations were stratified by sex, age, and number of dental amalgam surfaces.

**Results:**

The overall mean urinary mercury concentration varied between 0.12 μg Hg/L and 0.31 μg Hg/L or 0.13 μg Hg/g Cr and 0.40 μg Hg/g Cr. In general, females showed slightly higher mean urinary mercury levels than men. The overall 95^th^ percentile was 2.95 μg Hg/L, the 99^th^ percentile was 7.34E μg Hg/L, and the 99.9^th^ percentile was 17.45 μg Hg/L. Expressed as μg Hg/g Cr, the overall 95^th^ percentile was 2.57 μg Hg/g Cr, the 99^th^ percentile was 5.65 μg Hg/g Cr, and the 99.9^th^ percentiles was 12.14 μg Hg/g Cr. Overall, 98.2% of participants had urinary mercury levels below 7 μg Hg/L and 97.7% had urinary mercury levels below 5 μg Hg/g Cr. All data are estimates for the Canadian population. The estimates followed by the letter “E” should be interpreted with caution due to high sampling variability (coefficient of variation 16.6%-33.3%).

**Conclusions:**

The mean urinary mercury concentrations in the general Canadian population are significantly lower than the values considered to pose any risks for health.

## Background

Mercury occurs in nature as elemental, inorganic, and organic mercury (i.e., methylmercury) [1]. In terms of human exposure, each form has different absorption and excretion pathways and rates, different half-lives, and different adverse effects [2]. The general population is exposed mainly to methylmercury primarily through the consumption of contaminated fish and seafood. To a much lesser extent, the general population is exposed to inorganic and elemental mercury from sources such as dental amalgam [3].

Dental amalgam has been in use for more than 150 years. It provides advantages over other restorative materials in that it may be placed quickly in a wet field while providing high strength, durability, longevity, and marginal integrity; features that may help prevent recurrent decay. It is indicated in stress-bearing areas in posterior teeth, and is ideal when a patient’s oral hygiene is poor [4]. Low levels of elemental mercury vapours are released from an amalgam filling’s surface. *In vitro* studies have shown that the rate and amount of mercury released depends on the age and the condition of the amalgam restoration; the formation of a passive tarnish layer on the surface of the restoration is believed to be associated with a decrease in mercury vapour release [2]. The release of elemental mercury vapour is stimulated by chewing, tooth brushing, bruxism, and the ingestion of hot foods and liquids [5]. Based on the available literature, it has been estimated that 15.25% of the total mercury released from dental amalgam (i.e., elemental and inorganic) is absorbed; most inorganic mercury released from amalgam is excreted [6].

The current controversy surrounding the use of dental amalgam centers on the environmental pollution of dental waste, and on the safety of dental amalgam in individuals. Worldwide, dentistry has the smallest contribution to environmental mercury pollution (i.e., 0.04%-0.2%) [7]. In Canada, the use of amalgam separators in dental offices is expected to achieve a 95% national reduction in mercury release from dental amalgam waste discharge into the environment [8]. It has been reported [9] that starting in January 2008, Norway banned amalgam restorations due to their association with environmental mercury pollution. However, some argue that a worldwide ban of dental amalgam would have little or no environmental impact on total worldwide mercury pollution [7].

In individuals, a number of hypotheses suggest that mercury exposure from dental amalgam contributes to various diseases. Reviews of the clinical evidence do not support these hypotheses [5]. Importantly, the existing data also do not suggest that fetuses are at risk for adverse health effects due to maternal exposure to mercury vapours from dental amalgam [10].

Urinary mercury concentrations are the most accurate and widely used biomarker for assessing the absorbed dose that results from chronic mercury vapour exposure [11]. Urinary mercury concentrations can be expressed as μg Hg per gram creatinine (Cr) or μg Hg per liter of urine (L). Human Biomonitoring (HBM) values are derived on the basis of toxicological and epidemiological studies. The HBM-I-value represents the concentration of a substance in human biological material below which there is no risk for adverse health effects and, consequently, no need for action. The HBM-I is a verification or control value. The HBM-II value represents the concentration above which there is an increased risk for adverse health effects. The HBM-II value is the intervention or action level [11] (Table 1).

**Table 1:** Urinary mercury concentrations and health effects

**No risk for adverse health effects (HBM-I)**[11]	**Increased risk for adverse health effects (HBM-II)**[11]	**Onset of sub-clinical adverse effects on kidney function**[5]	**Onset of clinical mercurialism**[5]
***No action required***	***Action required***		
**5 μg Hg/g Cr**	**20 μg/g Cr**	**50 μg Hg/g Cr**	**100 μg Hg/g Cr**
*or*	*or*		
**7 μg Hg/L**	**25 μg/L**		

Previous studies of adults with dental amalgam fillings have found a positive and consistent correlation between the number of dental amalgam restorations and the mean urinary mercury concentration (mumc). Data from the 1999–2000 National Health and Nutrition Examination Survey (NHANES) indicate that women of childbearing age (16 to 49 years old) with 12 amalgam surfaces or less have an average of 0.81 μg Hg/g Cr [12]. This value was 6 times lower than the values accepted to pose no risks for health [11]. Non-occupationally exposed adults aged 30–49, with a mean number of 10.6 amalgam surfaces, had a mumc of 1.7 μg Hg/g Cr [13]. This value was 3 times lower than the values known to pose no risks for health [11].

One U.S. study of men aged 48–78, with an average of 19.9 amalgam surfaces had a mumc of 2.88 μg Hg/L. The study estimated that the urinary mercury increased by approximately 0.1 μg Hg/L for each surface with dental amalgam. For individuals with no amalgam fillings, the mumc was 0.70 μg Hg/L [14]. Individuals with amalgam restorations had a mumc 2.5 times lower than the values known to pose no risks for health [11].

In a seven-year Portuguese study of children aged eight to ten at baseline, the highest mumc reported was 3.2 μg Hg/g Cr. This level occurred during the second year of follow-up and declined progressively through year seven. The subjects had an average total of 19 amalgam surfaces at the end of the study period [15]. The highest mumc reported during the study period was 3.2 μg Hg/g Cr; this value was 1.5 times lower than the values known not to pose any risks for health [11].

In a five-year U.S. study of children ages six to ten at baseline, the mumc was 0.9 μg Hg/g Cr five years after amalgam placement. The average total number was 12 amalgam surfaces at the end of the study period. Children having only composite restorations had a mumc of 0.6 μg Hg/g Cr [16]. Children with amalgam restorations had a mumc 5.5 times lower than the values known not to pose any risks for health [11].

Finally, data from the 2003–2004 NHANES indicated an overall mumc of 0.447 μg Hg/L for people up to age 20 and over [17]. This value was almost 16 times lower than the values known to pose no risks for health [11].

With the recent 2007/09 Canadian Health Measures Survey (CHMS), there is now an opportunity to explore this issue within the Canadian context. The purpose of the present study is to determine the overall urinary mercury level in the Canadian general population, and to establish if there is a relationship between urinary mercury concentrations and the number of amalgam surfaces.

## Methods

### Sample

The CHMS is the most comprehensive national survey on physical health measures ever undertaken in Canada. The CHMS protocol was reviewed by the Health Canada Research Ethics Board, in compliance with the Helsinki Declaration; Certificate of Approval project file number REB-2005-0025. CHMS data collection occurred in two stages: (i) a household interview; and (ii) clinical examination. The household interviews collected information on socioeconomic status, health behaviors, and demographic characteristics. The clinical examination collected clinical measures of physical health (including urine spot samples and an oral health examination) during visits to the CHMS mobile examination center (see below) [18].

The CHMS provides national (not provincial) estimates for conditions with a prevalence of 10% or higher and a coefficient of variation of 16.5%. Canada was divided into 257 potential collection sites, each with a population of greater than 10,000. The region (British Columbia, Prairies, Ontario, Quebec, Atlantic) and urban/rural nature of each site was identified and then 15 sites were systematically selected in proportion to the size of their population. Within each site, households with known household composition (based on the 2006 census) were divided into six strata to obtain sufficient numbers of respondents in each of the targeted age groups. A random sample of households from each stratum was taken. Within a selected household, one or two respondents were selected. All five regions were represented. People living on Indian Reserves and on Crown Lands, institutional residents, full-time members of the Canadian Forces, and residents of certain remote regions or in areas with low population densities were excluded from the survey’s sampling frame [18].

Of the 8,772 households selected for the CHMS, 69.6% agreed to participate; 88.3% of them responded to the household interview, and of those, 84.9% visited the mobile examination center. The overall response rate was 51.7%. A comprehensive consent process was employed; participation was voluntary and respondents could opt out of any part of the survey at any time. The final CHMS sample size is 5,604 respondents and is representative of approximately 96.3% of the Canadian population [18]. This analysis is restricted to those with urine samples, and to the dentate population (having at least one natural tooth), excluding the edentulous population (having no natural teeth). This restriction reduces the usable sample size to 5,418.

### Data collection

In terms of the clinical oral health examination, the Canadian Forces supplied the dentists to conduct the examinations. All examiners were calibrated to World Health Organization standards. The first day at each new site was used for recalibration for all measures. All examiners achieved high agreement (Cohen’s Kappa ≥ 0.6) initially at all site locations. The clinical protocol was designed to collect tooth-specific information. All data were directly entered on a computer by a dental recorder at the time of the oral health examination at the mobile examination center (MEC). The MEC dental examining room was equipped with a portable chair (ADEC Portachair 3460), ceiling-mounted dental light, sterilizer (Tuttnauer Autoclave 1730 M), two operator stools, and computer for direct data entry. Examining instruments consisted of Williams Probe (Hu-Friedy).

PQW6), with markings at 1, 2, 3, 5, 7, 8, 9, 10 mm; mouth mirror (#4 head); cotton pliers; 2×2 cotton gauze; and cotton rolls [3].

To measure urinary mercury concentrations, urine spot samples were collected. Laboratory analysis of environmental chemicals and creatinine was performed at the Centre de toxicologie du Québec (CTQ) of L'Institut national de santé publique du Québec (INSPQ), Québec City. INSPQ followed standardized operating procedures that were developed for every assay and technique performed in their laboratory [3]. Replicate laboratory samples and commercial control samples were sent to the CHMS external laboratories to assess the accuracy and precision of laboratory testing. Field blanks were also sent to the CHMS external laboratories to ensure that samples were not being contaminated by the MEC environment and processes [3].

Inorganic mercury in urine was measured following an acid mineralization; the resulting solution was diluted and analyzed on the Flow Injection Mercury System (FIMS) module from Perkin Elmer (M-568) using cold vapour atomic absorption spectrometry. Ionized mercury was reduced to metallic mercury by the action of tin chloride. The volatile mercury formed was detected in the UV/VIS range [3].

Creatinine was measured using the colorimetric end-point Jaffe method. An alkaline solution of sodium picrate reacts with creatinine to form a red Janovski complex using Microgenics DRI “Creatinine-Detect” reagents (#917). The absorbance was read at 505 nm on a Hitachi 917 chemistry autoanalyzer (C-530) [3].

### Variables

The variables for this analysis included: sex (overall, female, male), age, and number of amalgam surface. The analysis was conducted for females, males and the two sexes combined, and for the following age intervals: 6–11 years, 12–19 years, 20–39 years, 40–59 years, 60–79 years, 6–79 years, and 16–49 years (females only).

### Data analysis

Under the Statistics Act, Statistics Canada is required to ensure respondent confidentiality. Therefore, estimates based on a small number of respondents are suppressed. Estimates of the geometric mean require at least 10 respondents. Estimates at the 95th percentile require at least 200 respondents [3].

The results are presented both as μg Hg/g Creatinine and μg Hg/L in order to facilitate comparison with other studies. Geometric means were determined because they are less influenced by extreme values, providing a better estimate of central tendency [19].

Statistical analyses were based on weighted data, and were descriptive. To account for survey design effects, standard errors, coefficients of variation and 95% confidence intervals were estimated using the bootstrap technique. Specifically, a survey weight is given to each person included in the final sample. This weight corresponds to the number of persons represented by the respondent for the entire population. When an estimate is produced, the survey weight must be used to ensure the estimate represents the population. If the survey weight is not used, the estimate represents only the sample [20].

In order to determine the quality of an estimate using the coefficient of variation (CV) or to calculate confidence intervals, the standard error of the estimate must be calculated. Since the CHMS uses a multi-stage survey design, there is no simple formula that can be used to calculate sampling variance. Instead, the bootstrap re-sampling method is used to take into account the sample design information and to easily obtain variance estimates. This method selects in each stratum, a simple random sample of (n-1) of the n first stage sampling units selected with replacement to form a replicate. In each replicate, the survey weight for each record in the (n-1) selected first stage sampling units is recalculated. These weights are then post-stratified according to demographic information in the same way as the survey weights in order to obtain the final bootstrap weights. This process is repeated 500 times for the CHMS [20].

All data were directly entered into a computer at the time of collection. For the oral health clinical examination, a detailed quality checking protocol was built into the data-entry program. With the extensive input of the Office of the Chief Dental Officer staff, Statistics Canada programmed entry values such that many areas of logical inconsistency were “greyed-out” and erroneous entries could not be made [20].

All analyses were conducted in-house by Statistics Canada. An experienced methodologist also verified the coding to produce all estimates in an effort to ensure reliability of the estimates. Furthermore, all numbers were verified prior to publication. The tables were formatted to display the CHMS findings in such a manner that both took advantage of the rich information and also would allow readers to compare the findings with those of the other publications [20].

## Results

The estimates are representative for the Canadian population (based on weighted data). All estimates accompanied by the letter “E” must be interpreted with caution due to high sampling variability; coefficient of variation 16.6% - 33.3%.

In the amalgam free group, selected percentiles (i.e., 95th, 99th,99.9th) and mumc estimates are provided for each age group (i.e., 6–11,12-19, 20–39, 49–59, 60–79, and 6–79 years old). In the Overall group, the number and percentage of people with urinary mercury concentrations above 7 μg Hg/L and 5 μg Hg/g Cr are presented in addition to the above (Tables 2 and 3). Note: as per Statistics Act, values could not be provided for categories with insufficient data for analysis.

**Table 2:** Selected percentiles, mean, number and percentage of people with urinary mercury concentrations above 7 μg Hg/L

**Characteristic**	**6-79 Years**	**6-11 Years**	**12-19 Years**	**20-39 Years**	**40-59 Years**	**60-79 Years**	**Women 16-49 Years**
**AMALGAM FREE**							
**95**^**th**^**Percentile**	0.89E	0.62	0.77E	0.93E	_	_	0.93E
**95% CI**	(0.5 - 1.28)	(0.41 - 0.82)	(0.44 -1.09)	(0.43 -1.43)			(0.41 - 1.45)
**99**^**th **^**Percentile**	2.31	1.38E	1.66	_	2.33E	2.32E	_
**95% CI**	(1.48 -3.13)	(0.81- 1.95)	(1.10– 2.22)		(1.15 -3.52)	(0.95 -3.7)	
**99.9**^**th **^**Percentile**	_	2.72E	_	_	4.28E	_	_
**95% CI**		(1.52 - 3.92)			(2.31 -6.24)		
**mumc**	0.10	0.08	0.10	0.11	0.11E	0.09	0.12
**95% CI**	(0.08 - 0.12)	(0.06 - 0.09)	(0.08 - 0.12)	(0.09 - 0.14)	(0.08 - 0.16)	(0.07 - 0.12)	(0.09 - 0.16)
**OVERALL**							
**95**^**th **^**Percentile**	2.95	1.92E	2.32	2.27	3.5	3.08	2.88
**95% CI**	(2.56 - 3.34)	(1.02 - 2.82)	(1.52 -3.11)	(1.86 -2.69)	(2.23 -4.78)	(2.59 - 3.57)	(1.88 - 3.88)
**99**^**th **^**Percentile**	7.34E	4.36E	4.64E	_	8.01E	7.31E	11.78E
**95% CI**	(4.27 -10.41)	(2.44 - 6.29)	(2.81 - 6.47)		(4.38 -11.65)	(3.89 - 10.73)	(3.82 - 19.75)
**99.9**^**th **^**Percentile**	17.45	7.59	_	17.27	17.35E	15.15E	18.14
**95% CI**	(14.22 - 20.68)	(5.17 - 10.01)		(12.03 -22.51)	(9.72 - 24.98)	(9.26 - 21.04)	(15.66 - 20.61)
**mumc**	0.22	0.12	0.15	0.20	0.31	0.22	0.23
**95% CI**	(0.19 - 0.25)	(0.10 - 0.14)	(0.12 - 0.18)	(0.17 - 0.23)	(0.26– 0.37)	(0.18 - 0.29)	(0.19 - 0.27)
**Number above**	49	2	3	13	19	12	20
**7 μg Hg/L**							
**% above**	<1.84	_	_	<3.26	<3.37	<2.35	<4.37
**7 μg Hg/L**							

Estimates representative for the Canadian general population (based on weighted data).

**Table 3:** Selected percentiles, mean, number and percentage of people with urinary mercury concentrations above 5 μg Hg/g Cr

**Characteristic**	**6-79 Years**	**6-11 Years**	**12-19 Years**	**20-39 Years**	**40-59 Years**	**60-79 Years**	**Women 16-49 Years**
**AMALGAM FREE**							
**95**^**th **^**Percentile**	0.89E	0.66	0.77E	0.85E	_	0.82E	_
**95% CI**	(0.46 - 1.32)	(0.46 - 0.86)	(0.44 - 1.09)	(0.28 -1.42)		(0.28 -1.36)	
**99**^**th **^**Percentile**	2.29E	1.35	1.66	_	2.84E	2.09E	_
**95% CI**	(1.06 - 3.51)	(0.91 - 1.78)	(1.10 - 2.22)		(1.04 - 4.63)	(1.18 - 2.99)	
**99.9**^**th **^**Percentile**	_	2.39E	_	_	3.32E	_	_
**95% CI**		(1.35 - 3.44)			(2.1 - 4.55)		
**mumc**	0.12	0.12	0.09	0.13	0.15E	0.14	0.16E
**95% CI**	(0.10 - 0.16)	(0.10 - 0.14)	(0.07 - 0.11)	(0.10 -0.18)	(0.10 - 0.22)	(0.10 - 0.18)	(0.11 - 0.24)
**OVERALL**							
**95**^**th **^**Percentile**	2.57	2.02	2.32	1.93	3.04	2.77	2.67
**95% CI**	(2.16 -2.97)	(1.33 - 2.7)	(1.52 - 3.11)	(1.56 -2.3)	(2.35 -3.73)	(2.15 -3.38)	(1.88 - 3.46)
**99**^**th **^**Percentile**	5.65	5.2E	4.64E	4.64E	5.9E	7.34	6.26E
**95% CI**	(3.66 - 7.63)	(2.36 - 8.04)	(2.81 - 6.47)	(2.55 - 6.72)	(3.72- 8.07)	(5.17- 9.52)	(3.83- 8.69)
**99.9**^**th **^**Percentile**	12.14	10.04	_	12.56E	11.59	_	12.87E
**95% CI**	(10.03– 14.25)	(7.00 - 13.08)		(5.65 - 19.48)	(8.77 - 14.42)		(6.7 - 19.03)
**mumc**	0.26	0.18	0.13	0.22	0.40	0.31	0.31
**95% CI**	(0.23 - 0.30)	(0.15 - 0.22)	(0.11 - 0.15)	(0.19 - 0.25)	(0.33 - 0.48)	(0.24 - 0.41)	(0.27 - 0.36)
**Number above**	71	15	3	10	28	15	19
**5 μg Hg/g Cr**							
**% above**	<2.32	<2.64	_	<1.77	<4.58	<2.88	<4.74
**5 μg Hg/g Cr**							

Estimates representative for the Canadian general population (based on weighted data).

### People 6–79 years old

The mumc for people with the largest number of amalgam surfaces 2.45E μg Hg/g Cr, 95% CI (1.31 - 4.59) (Tables 4 and 5). There were 13 individuals (i.e., 4 females and 9 males) with 46–65 amalgam surfaces. When measured as μg Hg/L, 49 people 6–79 years of age in the CHMS sample, representing approximately 536,498 Canadians, or less than 1.84% of the population (6–79 years of age) were identified as having urinary mercury levels above HBM I values. Estimated as μg Hg/g Cr, 71 people in the CHMS sample, representing approximately 676,454 Canadians, or less than 2.32% of the population (6–79 years of age) had urinary mercury levels above HBM I values (Tables 2 and 3).

**Table 4:** Mean urinary mercury concentrations (μg Hg/L) in people 6 to 79 years of age

**Characteristic**	**Number of amalgam surfaces**										**Number of amalgam surfaces**									
	**All**		**0 Surfaces**		**1-5 Surfaces**		**6-10 Surfaces**		**11-15 Surfaces**		**16-20 Surfaces**		**21-25 Surfaces**		**26-30 Surfaces**		**31-45 Surfaces**		**46-65 Surfaces**	
	**Mean**	**95% CI**	**Mean**	**95% CI**	**Mean**	**95% CI**	**Mean**	**95% CI**	**Mean**	**95% CI**	**Mean**	**95% CI**	**Mean**	**95% CI**	**Mean**	**95% CI**	**Mean**	**95% CI**	**Mean**	**95% CI**
**All**	0.22	0.19-0.25	0.10	0.08-0.12	0.19	0.14-0.25	0.31	0.26-0.38	0.39	E 0.26-0.58	0.65	0.50-0.85	0.84	E 0.56-1.27	1.78	E 1.23-2.58	1.92	E 1.00-3.69	F	F
**Female**	0.22	0.19-0.25	0.10	0.08-0.12	0.18	0.14-0.23	0.28	0.22-0.36	0.42	E 0.26-0.68	0.74	0.53-1.03	0.72	E 0.35-1.47	1.58	E 0.93-2.69	2.16	E 1.13-4.14	F	F
**Male**	0.22	0.19-0.25	0.10	0.08-0.12	0.20	E 0.13-0.30	0.35	0.27-0.45	0.37	E 0.24-0.56	0.58	E 0.35-0.95	1.03	E 0.68-1.56	2.13	E 1.39-3.28	F	F	2.55	E 1.45-4.47

Estimates representative for the Canadian general population (based on weighted data).

Mean = Geometric Mean.

E Interpret with caution (high sampling variability; coefficient of variation 16.6%-33.3%).

F Estimate not provided because of extreme sampling variability or small sample size.

**Table 5:** Mean urinary mercury (μg Hg/g Cr) in people 6 to 79 years of age

**Characteristic**	**Number of amalgam surfaces**										**Number of amalgam surfaces**									
	**All**		**0 Surfaces**		**1-5 Surfaces**		**6-10 Surfaces**		**11-15 Surfaces**		**16-20 Surfaces**		**21-25 Surfaces**		**26-30 Surfaces**		**31-45 Surfaces**		**46-65 Surfaces**	
	**Mean**	**95% CI**	**Mean**	**95% CI**	**Mean**	**95% CI**	**Mean**	**95% CI**	**Mean**	**95% CI**	**Mean**	**95% CI**	**Mean**	**95% CI**	**Mean**	**95% CI**	**Mean**	**95% CI**	**Mean**	**95% CI**
**All**	0.26	0.23-0.30	0.12	0.10-0.16	0.22	0.17-0.28	0.36	0.30-0.44	0.52	0.38-0.71	0.79	0.63-0.99	1.09	0.80-1.49	1.91	1.49-2.46	2.12	E 1.21-3.71	2.45	E 1.31-4.59
**Female**	0.33	0.29-0.37	0.15	0.12-0.20	0.25	0.20-0.32	0.44	0.35-0.55	0.68	0.49-0.97	0.99	0.78-1.27	1.24	E 0.77-1.99	2.21	E 1.49-3.27	2.78	E 1.75-4.43	F	F
**Male**	0.21	0.18-0.25	0.10	0.08-0.13	0.19	0.14-0.26	0.31	0.24-0.39	0.41	0.28-0.58	0.63	E 0.41-0.97	0.93	0.68-1.27	1.54	1.11-2.13	1.43	E 0.75-2.73	F	F

Estimates representative for the Canadian general population (based on weighted data).

Mean = Geometric Mean.

E Interpret with caution (high sampling variability; coefficient of variation 16.6%-33.3%).

F Estimate not provided because of extreme sampling variability or small sample size.

### Children 6–11 years of age

Children with the largest recorded number of amalgam surfaces (i.e., 26–30) had a mumc of 4.77E μg Hg/g Cr, 95% CI (2.43 - 9.35). Only 2 children (i.e., one male and one female) had 26–30 amalgam surfaces. When measured as μg Hg/g Cr, 2 children 6–11 years of age in the CHMS sample were identified as having urinary mercury levels above HBM I values. Estimates of the Canadian population represented and the corresponding percentages could not be provided due to insufficient data for analysis. Estimated as μg Hg/g Cr, 15 children in the CHMS sample, representing approximately 57,035 Canadians, or less than 2.64% of the child population (6–11 years of age) had urinary mercury levels above HBM I values (Tables 2 and 3).

### Adolescents 12–19 years of age

Adolescents with the largest number of amalgam surfaces (i.e., 16–20) for which the mumc could be determined (females), had a mumc of 3.79E μg Hg/L, 95% CI (1.89 - 7.62) or 2.39E μg Hg/g Cr, 95% CI (1.42 - 4.01). There were7 adolescents (i.e., 3 females and 4 males) with 16–20 amalgam surfaces. Three adolescents in the CHMS sample had urinary mercury levels above HBM I values. Estimates of the Canadian population represented and the corresponding percentages could not be provided due to insufficient data for analysis (Tables 2 and 3).

### Young adults 20–39 years old

People in this age group with the largest number of amalgam surfaces (i.e., 46–65) had a mumc of 0.84 μg Hg/L, 95% CI (0.81 - 0.87) or 2.03 μg Hg/g Cr, 95% CI (2.030 - 2.034). There were 2 individuals (i.e., one female and one male) with 46–65 amalgam surfaces. When measured as μg Hg/L, 13 people 20–39 years of age in the CHMS sample, representing approximately 293,798 Canadians, or less than 3.26% of the population (20–39 years of age) were identified as having urinary mercury levels above HBM I values. Estimated as μg Hg/g Cr, 10 people in the CHMS sample, representing approximately 159,516 Canadians, or less than 1.77% of the population (20–39 years of age) had urinary mercury levels above HBM I values (Tables 2 and 3).

### Adults 40–59 years old

People with the largest number of amalgam surfaces (i.e., 46–65) had a mumc of 3.34E μg Hg/g Cr, 95% CI (1.72 - 6.50) (females). There were 4 individuals (i.e., 2 females and 2 males) with 46–65 amalgam surfaces. When measured as μg Hg/L, 19 people 40–59 years of age in the CHMS sample, representing approximately 328,642 Canadians, or less than 3.37% of the population (40–59 years of age) were identified as having urinary mercury levels above HBM I values. Estimated as μg Hg/g Cr, 28 people in the CHMS sample, representing approximately 446,642 Canadians, or less than 4.58% of the population (40–59 years of age) had urinary mercury levels above HBM I values (Tables 2 and 3).

### Older adults 60–79 years old

Older adults with largest number of amalgam surfaces (i.e., 46–65) also had the largest mumc, measured at 2.78E μg Hg/L, 95% CI (1.77 - 4.37) or 3.00E μg Hg/g Cr, 95% CI (1.82 - 4.95). There were 7 individuals (i.e., 1 female and 6 male) with 46–65 amalgam surfaces. When measured as μg Hg/L, 12 people 60–79 years of age in the CHMS sample, representing approximately 115,519 Canadians, or less than 2.35% of the population (60–79 years of age) were identified as having urinary mercury levels above HBM I values. Estimated as μg Hg/g Cr, 15 people in the CHMS sample, representing approximately 141,572 Canadians or less than 2.88% of the population (60–79 years of age) had urinary mercury levels above HBM I values (Tables 2 and 3).

### Women of childbearing Age (16–49 years old)

The mumc for women with the largest number of amalgam surfaces (i.e., 31–45) was 3.86E μg Hg/L, 95% CI (1.98 - 7.51) or 2.88E μg Hg/g Cr, 95% CI (1.65 - 5.03). There were 27 women with 31–45 amalgam surfaces. When measured as μg Hg/L, 20 women 16–49 years of age in the CHMS sample, representing less than 4.37% of the Canadian female population (16–49 years of age), were identified as having urinary mercury levels above HBM I values. Estimated as μg Hg/g Cr, 19 women in the CHMS sample, representing less than 4.74% of the Canadian female population (16–49 years of age) had urinary mercury levels above HBM I values (Tables 2 and 3). The data supplied by Statistics Canada did not include an estimate of the total Canadian female population in the 16 to 49 age group so the total population represented by the 20 and 19 women cannot be provided.

### Trends

In general, females tend to have slightly larger urinary mercury concentrations than males. The difference in the overall mumcs between the two sexes increases from 0.01 μg Hg/g Cr in children 6–11 years of age to 0.2 μg Hg/g Cr in adults 40–59 years of age. For the selected percentiles, the urinary mercury concentrations increase with the number of dental amalgam surfaces. Among the selected age groups (i.e., 6–11, 12–19. 20–39, 40–59, and 60–79 years) at least 96.63% - 98.16% had urinary mercury levels below 7 μg Hg/L, and at least 95.42% - 98.23% had urinary mercury levels below 5 μg Hg/g Cr (HBM-I values). At most, 3.37% - 1.84% and 4.58% - 1.77% respectively had estimates that should be verified by further measurements (between HBM-I and HBM-II) [11].

## Discussion

In Canada, the mumc in people with or without dental amalgam restorations are well below the levels associated with any health risks. In general, the mumcs tend to increase with the number of amalgam surfaces, and appear to be influenced by age and sex. On average, females showed slightly higher levels of urinary mercury than males. These observations are consistent with the findings of other researchers [21]. However, further research is needed to explore the linear and nonlinear relationship between urinary mercury concentrations and the number of amalgam surfaces.

For the same exposure level, organic mercury from food results in an absorption approximately six-times greater than the absorption of inorganic mercury from dental amalgam restorations [6]. The CHMS results indicate that total mercury in blood is well below the guidance levels. More importantly, estimates for the mean inorganic mercury in blood could not be provided since more than 40% of the samples were below the level of detection [3]. It is important to note that urinary excretion of mercury is a measure of the body’s ability to eliminate this substance following exposure. The presence of measurable amounts of mercury in urine does not imply that adverse health effects will occur [3]. The results of our investigation show that the vast majority of the Canadian general population (i.e., 95.42% - 98.23%) has urinary mercury concentrations below the levels associated with any health risks.

Fish consumption can elevate mumcs; once ingested, some of the methylmercury from fish is de-methylated to inorganic mercury, and excreted in urine [3,22-25]. The mumcs are also influenced by the age of the amalgam restorations and bruxism [2,5,26]. Since the true size of the influence exerted by other sources of mercury was not accounted for, the exact contribution of dental amalgam alone to the observed urinary mercury levels in the Canadian general population could not be assessed.

Elemental, inorganic, and organic mercury compounds have specific half-lives and metabolic pathways. The high sampling variability reflects mercury ubiquity and its diverse metabolic pathways, which influence the uptake, distribution, accumulation patterns and excretion rates. The same baseline mercury exposure was assumed for all survey participants (e.g., with and without amalgam restorations); it is unknown if it was a correct assumption. For instance, one might argue that individuals with amalgam restorations have a different level of exposure from other sources than people without such restorations.

The results of this research indicate that the overall mumc in the Canadian general population is two times lower than the overall mumc reported by the 2003–2004 NHANES [17]. Further, the overall mumc in Canadian women of childbearing age is more than two times lower than the mumcs reported by other researchers for the same age and gender population subgroup [12].

In a number of case studies, Barregard *et al.* found that people with bruxism and/or who were heavy gum chewers had urinary mercury levels ranging from 25 μg Hg/g Cr to 54 μg Hg/g Cr [27]. Such urinary mercury levels are in the range commonly observed among occupationally exposed individuals [27]. The results of our study show that the highest overall mumc was 0.40 μg Hg/g Cr, 95% CI (0.33 - 0.48); the highest 99.9^th^ percentile estimate provided was 12.87E μg Hg/g Cr, 95% CI (6.7 - 19.03). Although high, this 99.9^th^ percentile value is not associated with adverse health effects. Yet it indicates that some people require further investigation [11]. Additionally, people with bruxism and/or people with intense chewing gum use should be identified and advised about their risk of increased mercury exposure from their dental amalgam restorations due to these habits.

In people without dental amalgam, the difference between the estimates provided by Barregard *et al.*[27] and the results of our study indicate that exposure to mercury from sources other than dental amalgam can vary significantly between people. As mentioned, the factors that contribute to measured blood and urine levels include the quantity entering the body through all routes of exposure, absorption rates, distribution to various tissues in the body, metabolism, and excretion of the chemical and/or its metabolites from the body. These processes are dependent on both the characteristics of the chemical and the characteristics of the individual, such as age, diet, gender, health status, smoking or a history of smoking, medication and its side effects. For these reasons, the way in which a chemical will act in the body will differ among individuals and cannot be predicted with certainty [3].

Although most studies on the subject report mumcs, it is important to look beyond the average [28]. Selected percentiles provide valuable information about the upper tail. The estimates of our study show that some people have urinary mercury concentrations between HBM I and HBM II. For these people a reassessment is indicated. In case the urinary mercury levels remain high, further research is needed to identify, quantify and decrease mercury exposure [11]. Most importantly, all the estimates provided, whether mumc or selected percentiles, are below the urinary mercury levels known to be associated with the onset of sub-clinical adverse effects on kidney function.

A recent study explored various scenarios of mercury exposure and risks from dental amalgam in the US population. According to the worst case scenario more than 60 million Americans would exceed the Hg reference exposure level established by the US Environmental Protection Agency [29]. However, it is important to mention that not every aspect of Hg exposure, toxicity or pharmacokinetics was considered for the scenarios explored [29]. For instance, methylmercury exposure and its contribution to urinary mercury concentration was not addressed. The author further indicates that the exposure estimates are consistent with previous estimates prepared for Health Canada in 1995. At that time, Health Canada concluded that the amalgam risk assessment conducted by the author was based on too many assumptions and that the safety factor was chosen arbitrarily, arguably rendering the estimates provided as inappropriate [5].

While a worldwide ban of dental amalgam will not impact on total worldwide mercury pollution [7], a ban on amalgam arguably would lead to increases in dental care expenditure. First, the materials that substitute for amalgams (i.e., composite resins and cast restorations) are more expensive per restoration. Second, composites do not last as long as amalgams and require more frequent replacement [30]. Each time a restoration is replaced additional natural tooth structure is lost, requiring larger and larger new restorations [31]. The replacement restorations are often more complex as well, and thus more expensive. In addition to the direct expenditures, there will also be a significant increase in the indirect expenditures associated with patient time and travel costs for more dental visits.

Disease rates are higher in the poorest and the most vulnerable populations; such cost increases would arguably concentrate in these already disadvantaged groups. Using population projections to obtain national estimates of dental amalgam use, and the dental component of the Consumer Price Index to estimate the annual rate of change in fees, it has been estimated that the first-year of banning dental amalgam in the entire population in the U.S. would lead to an $8.2 billion increase in direct dental expenditures alone [30].

Health Canada points out that current evidence does not indicate that dental amalgam is causing illness in the general population [32]. Also, according to the Canadian Dental Association (CDA), the risks associated with the use of dental amalgam appear to be limited, and the benefits to patients are known to be large [33]. The CDA’s confidence in this restorative material is based on the observation of patients over the course of 150 years of using dental amalgam [33]. Recent advances, such as the development of amalgam bonding techniques, have also made dental amalgam even more advantageous as a restorative material [33].

Importantly, dental amalgams are usually replaced with composite resin restorations, which are a source of BPA [3]. The European Chemicals Bureau has classified BPA as a reproductive toxicant, that is, a substance that causes concern for human fertility. The key effects considered by Health Canada as appropriate departure points for the characterization of risk to human health from exposure to BPA involve the liver and reproductive system, including effects on fertility and development [3]. BPA is considered an environmental estrogen that can stimulate cellular responses at very low concentrations. The potential role of BPA and other environmental estrogens in the prevalence of obesity and related metabolic diseases, as well as certain types of cancer, is under intensive debate and investigation. Health Canada has conducted a scientific screening assessment of the impact of human and environmental exposure to BPA and determined that it is of concern to human health and the environment as per the criteria set out under the Canadian Environmental Protection Act, 1999 [3].

## Conclusions

For the vast majority of Canadians, the mean urinary mercury concentrations are below a level of concern regardless of the number of amalgam surfaces they have. It is arguable that from a population health standpoint, dental amalgam remains a safe restorative material.

## Consent

Written informed consent was obtained from the patient’s guardian/parent/next of kin for the publication of this report and any accompanying images.

## Abbreviations

CHMS: Canadian health measure survey; CTQ: Centre de toxicologie du Québec; Cr: Creatinine; Hg: Mercury; INSPQ: L'Institut national de santé publique du Québec; NHANES: National health and nutrition examination survey; Mumc: Mean urinary mercury concentration; Mg Hg/L: Micrograms mercury per liter (in urine); Mg Hg/g: Cr micrograms mercury per gram creatinine (in urine).

## Competing interests

The authors declare that they have no competing interests.

## Authors’ contributions

AN participated in the design of the study, results interpretation and helped to draft the manuscript. HA conceived of the study, and participated in its design and coordination and helped to draft the manuscript. CQ participated in the design of the study and helped to draft the manuscript. All authors read and approved the final manuscript.

## Authors’ information

AN, DDS, MSc, FRCD(C), HA, DDS, MPH, Membership Services Coordinator, Alberta Dental Association.

CQ, DMD, MSc, PhD, FRCD(C) Assistant Professor and Director, Specialty Training Program (Dental Public Health) Dept. of Biological and Diagnostic Sciences/Community Dentistry, University of Toronto.

## Pre-publication history

The pre-publication history for this paper can be accessed here:

http://www.biomedcentral.com/1472-6831/13/44/prepub
